# Drought yield index to select high yielding rice lines under different drought stress severities

**DOI:** 10.1186/1939-8433-5-31

**Published:** 2012-10-04

**Authors:** Anitha Raman, Satish Verulkar, Nimai Mandal, Mukund Variar, V Shukla, J Dwivedi, B Singh, O Singh, Padmini Swain, Ashutosh Mall, S Robin, R Chandrababu, Abhinav Jain, Tilatoo Ram, Shailaja Hittalmani, Stephan Haefele, Hans-Peter Piepho, Arvind Kumar

**Affiliations:** 1 International Rice Research Institute (IRRI), DAPO Box 7777, Metro Manila, Philippines; 2 Indira Gandhi Krishi Vishwavidyalaya (IGKV), Raipur, India; 3 Central Rainfed Upland Rice Research Station (CRURRS), Hazaribag, India; 4 Narendra Dev University of Agriculture and Technology (NDUAT), Faizabad, India; 5 Birsa Agricultural University (BAU), Ranchi, India; 6 Central Rice Research Institute (CRRI), Cuttack, India; 7 Tamil Nadu Agricultural University (TNAU), Coimbatore, India; 8 Barwale Foundation (BF), Hyderabad, India; 9 Directorate of Rice Research, Hyderabad, India; 10 University of Agricultural Sciences, Bangalore, India; 11 Bioinformatics Unit, Universitaet Hohenheim, 70593 Stuttgart, Germany

**Keywords:** Rice, Drought, Genotype x environment interaction, Drought yield index, Mixed model

## Abstract

**Background:**

Drought is the most severe abiotic stress reducing rice yield in rainfed drought prone ecosystems. Variation in intensity and severity of drought from season to season and place to place requires cultivation of rice varieties with different level of drought tolerance in different areas. Multi environment evaluation of breeding lines helps breeder to identify appropriate genotypes for areas prone to similar level of drought stress. From a set of 129 advanced rice (*Oryza sativa* L.) breeding lines evaluated under rainfed drought-prone situations at three locations in eastern India from 2005 to 2007, a subset of 39 genotypes that were tested for two or more years was selected to develop a drought yield index (DYI) and mean yield index (MYI) based on yield under irrigated, moderate and severe reproductive-stage drought stress to help breeders select appropriate genotypes for different environments.

**Results:**

ARB 8 and IR55419-04 recorded the highest drought yield index (DYI) and are identified as the best drought-tolerant lines. The proposed DYI provides a more effective assessment as it is calculated after accounting for a significant genotype x stress-level interaction across environments. For rainfed areas with variable frequency of drought occurrence, Mean yield index (MYI) along with deviation in performance of genotypes from currently cultivated popular varieties in all situations helps to select genotypes with a superior performance across irrigated, moderate and severe reproductive-stage drought situations. IR74371-70-1-1 and DGI 75 are the two genotypes identified to have shown a superior performance over IR64 and MTU1010 under all situations.

**Conclusion:**

For highly drought-prone areas, a combination of DYI with deviation in performance of genotypes under irrigated situations can enable breeders to select genotypes with no reduction in yield under favorable environments compared with currently cultivated varieties. For rainfed areas with variable frequency of drought stress, use of MYI together with deviation in performance of genotypes under different situations as compared to presently cultivated varieties will help breeders to select genotypes with superior performance under all situations.

## Background

Rainfed lowland rice ecosystems are highly variable and unpredictable in nature (Yoshida 1977). Multiple abiotic stresses such as unfavorable soil conditions, regional weather patterns, topography, pests and weeds all contribute to the complexity of the ecosystem. The worldwide harvested area of rainfed lowland rice is estimated to be 46 to 48 million hectares. Of this, 90% is in South and Southeast Asia. Rice farming in these rainfed areas is risk-prone. Yields remain low, about 1.5 to 2.5 t ha^-1^ in most areas. The income of most farmers is low and they are challenged by erratic yields. In Asia, about 50% of all the rice land is rainfed and although rice yields in irrigated ecosystems have doubled and tripled over the past 30 years, only modest gains have occurred in rainfed rice systems (Fischer et al. 2003). The water supply in rainfed areas principally comes from rainfall. Uncertainty in the timing of rainfall and variability in its intensity and its distribution cause either flood or drought stress in rainfed lowland rice production ecosystem.

Drought is the major constraint to productivity and the cause of yield instability in rainfed lowlands. At least 23 million ha of rice area in Asia are estimated to be drought-prone (Pandey et al. 2005). Severe and regular droughts affect mainly eastern India, northeast Thailand and parts of Myanmar and Laos. In India, the rainfed lowland rice area is about 20.4 million hectares, which accounts for 32.4% of the total area under rice in the country. Out of the total of 20.4 million ha of rainfed rice area, approximately 7.3 million ha of lowland area are drought-prone (Pandey and Bhandari 2008). The timing of drought, early season, mid-season or terminal stage, has a major influence on how much yield loss occurs (Fischer et al. 2003). Drought patterns also differ among locations and among years, and in some years no drought occurs during the growing season. Therefore, poverty reduction strategies in rainfed areas must focus on stabilizing yields, that is, on breeding varieties with improved yield under drought stress as well as good response to irrigated conditions. To a farmer’s eyes, a drought-resistant cultivar is one that yields better than any other available cultivar particularly under water-limited conditions (Blum 2006). The objective of a drought-tolerance breeding program is to select varieties that outperform currently available varieties in the target population of environments (TPE). The TPE is the future set of drought-prone environments in which the varieties developed by the breeding program will be grown. The environments in the TPE vary in predictable ways such as annual rainfall patterns, toposequence, soil type and farmers practices and in unpredictable ways such as random drought or disease incidence (Fischer et al. 2003). Variability among environments within TPE (locations and seasons) is particularly common in rice.

The ability of crop cultivars to perform reasonably well in drought-stressed environments is paramount for stability of production. The relative yield performance of genotypes in drought-stressed and non-stressed environments can be used as an indicator to identify drought-resistant varieties in breeding for drought-prone environments. Several drought indices have been suggested on the basis of a mathematical relationship between yield under drought conditions and non-stressed conditions. These indices are based on either drought resistance or drought susceptibility of genotypes.

Let (*Y*_*i*_)_*S*_ denote the yield of the i^th^ genotype under stress, (*Y*_*i*_)_*NS*_ the yield of the i^th^ genotype under non-stress (i.e., irrigated) conditions and *y*_*S*_ and *y*_*NS*_ the mean yields of all genotypes evaluated under stress and non-stress conditions, respectively.Rosielle and Hamblin (1981) defined stress tolerance (TOL) as the differences in yield between the stress and non-stress environments, i.e., $TOL={\left(Y_{i}\right)}_{\text{NS}}-{\left(Y_{i}\right)}_{S}$. Higher values of TOL indicate susceptibility of a given cultivar.Hossain et al. (1990) defined mean productivity index (MPI) as the average of (*Y*_*i*_)_*NS*_ and (*Y*_*i*_)_*S*_. This index has an upward bias when the differences between non-stress and stress conditions are large and it favors genotypes with higher yield potential and lower stress tolerance. Higher values mean a higher rate of productivity. Mean relative performance is calculated as $MRP=\frac{{\left(Y_{i}\right)}_{S}}{Y_{S}}+\frac{{\left(Y_{i}\right)}_{\text{NS}}}{Y_{\text{NS}}}$ and relative efficiency is given by $REI=\frac{{\left(Y_{i}\right)}_{S}}{Y_{S}}*\frac{{\left(Y_{i}\right)}_{\text{NS}}}{Y_{\text{NS}}}$,Ramirez Vallejo and Kelly (1998) computed the geometric mean of productivity (GMP), which is the square root of the product of yield under stress and yield under non-stress: $GMP=\sqrt{{\left(Y_{i}\right)}_{S}*{\left(Y_{i}\right)}_{\text{NS}}}$. This index is suitable when the breeding objective is directed toward testing performance under favorable and stress conditions, taking into consideration variability in drought intensity over environment and years.Fernandez (1992) defined a stress tolerance index as $STI=\frac{\left({\left(Y_{i}\right)}_{\text{NS}}*{\left(Y_{i}\right)}_{S}\right)}{Y_{\text{NS}}^{2}}$, which can be used to identify genotypes that produce high yield under both stress and non-stress conditions. A high value of STI implies higher tolerance of stress. A stress susceptibility index (SSI) that assesses the reduction in yield caused by unfavorable compared with favorable environments was suggested byFischer and Maurer (1978). SSI is expressed by $SSI=\frac{\left(1,-,\frac{{\left(Y_{i}\right)}_{S}}{{\left(Y_{i}\right)}_{\text{NS}}}\right)}{SI}$, SI, the stress intensity is estimated as $SI=1-\frac{Y_{S}}{Y_{\text{NS}}}$. Lower SSI values indicate lower differences in yield across stress levels, in other words, more resistance to drought. A modified formula for Schneider’s stress severity index (Schneider et al. 1997) is defined bySingh et al. (2011) as $SSSI=\left(1-\frac{{\left(Y_{i}\right)}_{S}}{{\left(Y_{i}\right)}_{\text{NS}}}\right)-\left(1-\frac{Y_{S}}{Y_{\text{NS}}}\right)$. The SSSI estimates the relative tolerance for yield reduction of a genotype relative to the population mean reduction in grain yield response due to stress. Selections based on these indices were carried out by many authors (Pantuwan et al. 2002,Ouk et al. 2006,Golabadi et al. 2006, Sio-SeMardeh et al. 2006,Talebi et al. 2009,Khayatnezhad et al. 2010,Nouri et al. 2011,Singh et al. 2011).

Phenology is important in determining grain yield response also because quick maturing cultivars often escape from severe stress while late maturing cultivars may be affected by terminal stress (Singh et al. 1996). Research on genetic variation in grain yield of pearl millet under post flowering indicated that as much as 50% of the total variation in grain yield under stress is explained by yield potential and time of flowering.Bidinger et al. (1987) calculated a drought response index based on a regression model to quantify the remaining part of the variation associated with tolerance / susceptibility and to identify traits linked to tolerance. The following quadratic equations were used to develop stress indices for lines in advanced screening. Mid-season stress is defined by the regression equation ${\hat{Y}}_{s}=a+b_{1}\left(Y_{c}\right)+b_{2}\left(bl\right)+b_{3}\left(bl^{2}\right)$and terminal stress by ${\hat{Y}}_{s}=a+b_{1}\left(Y_{c}\right)+b_{2}\left(bl\right)$, where $\hat{Y_{s}}$is the regression estimate of the stress yield, *Y*_*s*_ is the measured stress yield, *Y*_*c*_ is the non-stress control yield, *bl* the days to flowering under non-stress, *a* the intercept and *b*_1_, *b*_2_ and *b*_3_ the regression coefficients. The drought index is then given by $\frac{\left(Y_{s}-{\hat{Y}}_{s}\right)}{S.E.\left({\hat{Y}}_{s}\right)}$, where *S*.*E*. indicates the standard error. Attention is focused only on those cultivars with indices of less than −1.3 or more than +1.3. These represent the upper and lower 10% of the normal distribution of the indices. FollowingBidinger et al. (1987),Slim and Saxena (1993) used a DRI to describe the response of individual chickpea genotypes to drought conditions and fitted multiple regression of stressed grain yield on unstressed grain yield and days to flowering: ${\hat{Y}}_{0}\;=\;a\;-\;bF\;+\;CY_{i}$. Here, $\hat{Y_{0}}$is the regression estimate of yield under drought, *Y*_*i*_ is the yield potential, *F* is the number of days to flowering under non-stress, *b* and *C* are the regression coefficients and *a* is the intercept. The drought response index is then given by $DRI=\frac{\left(Y_{0}-{\hat{Y}}_{0}\right)}{S.E.\left({\hat{Y}}_{0}\right)}$, where S.E indicates the standard error. (Garrity and O’Toole 1995) proposed a field screening method for reproductive phase drought resistance in rice based on the drought index suggested byBidinger et al. (1982) to adjust for genotypic variation in phenlogy before genotypic difference in drought tolerance is estimated.

Under severe stress, yield reduction in rice is 65-85% compared with that in non-stress conditions (Kumar et al. 2008). Rice is particularly sensitive to drought stress during reproductive growth, even under moderate drought stress (Hsiao 1982,O’Toole 1982). In rice, moderate stress can be broadly characterized by a 31 to 64% loss in grain yield as compared with non-stress conditions (Kumar et al. 2008). The objective of our study is to develop a drought yield index (DYI) that takes into account yield under both moderate and severe drought stress for the identification of breeding lines with superior performance over current cultivated varieties. The mean yield index (MYI) is proposed for rainfed areas where, in different years or during different growth stages of the crop in the same year, water availability fluctuates between a normal supply of water due to favorable rain and the occurrence of mild and moderate to severe drought stress based on the duration of days without rain during the cropping season. The MYI enables breeders to identify breeding lines with superior performance over current varieties under all situations.

## Methods

The genotypes evaluated in this study were 134 advanced breeding lines of 105–120 days of maturity duration generated from crosses of popular high-yielding varieties with a diverse array of donors for drought tolerance, and traditional drought-tolerant landraces. The entries were tested under a diverse set of conditions that ranged from favorable irrigated conditions (non-stress) to conditions with moderate to severe reproductive-stage drought in drought-prone eastern India. The lines were tested at three locations, Raipur, Hazaribag and Faizabad, in relatively similar shallow rainfed drought-prone ecosystems, from 2005 to 2007. At all sites, the experiments were planted under irrigated control and reproductive-stage drought stress. Experiments were planted in alpha lattice designs with three replicates. From the set of 134 genotypes, a subset of 39 genotypes (Table 1) that were evaluated in two or more years under non-stress, moderate stress and severe stress conditions in 16 environments at three sites was selected for analysis. A site-year-stress-level cross combination is referred to as a trial. The details of trials are presented in Table 2. For each drought-stress experiment, an irrigated control (non-stress) trial was planted at each of the locations. In non-stress experiments, standing water was maintained at each site from transplanting to 10 days before maturity by providing water by rain or by supplementary irrigation through a pump as and when required. The reproductive-stage drought-stress experiments were irrigated like the non-stress experiments by keeping standing water up to 28 days after transplanting. Thereafter, the stress fields were drained to allow them to dry and for stress to develop. The stress experiments were not provided with any supplemental irrigation after drainage even if the stress was very severe. This has been reflected in the difference in mean yield of the stress trials at different sites (Table 2). Some trials did not achieve the targeted stress levels due to rains during the wet season and were not exposed to the desired severity of drought stress. Therefore, after harvest, each stress trial was classified for observed stress intensity as moderately stressed if the yield reduction compared to the irrigated control trial was 31–65%, and severely drought-stressed if the yield reduction surpassed 65%.

**Table 1:** **Genotype mean yield and index values of breeding lines for the drought yield index** (**DYI**)

Breeding line	Non- stress	Moderate stress			Severe stress		
	Mean	Mean	p-value	Index	Mean	p-value	Index
Annada	4.14	2.89	0.15	1.43	1.66	0.001	2.49
ARB 2	4.33	2.98	0.11	1.45	1.71	0.001	2.53
ARB 3	4.82	3.00	0.04	1.61	1.83	0.001	2.63
ARB 4	4.27	2.73	0.06	1.57	1.86	0.004	2.30
ARB 5	4.19	2.90	0.12	1.44	1.89	0.006	2.22
ARB 6	4.64	2.70	0.02	1.72	1.73	0.001	2.69
ARB 7	4.24	3.00	0.15	1.41	2.05	0.017	2.07
ARB 8	4.47	3.35	0.23	1.33	2.19	0.019	2.05
Baranideep	4.61	2.87	0.06	1.60	1.66	0.000	2.78
CB 0-15-24	4.38	2.89	0.06	1.51	2.01	0.003	2.18
CB 2-458	4.65	2.40	0.01	1.94	1.40	0.000	3.32
DGI 237	4.28	2.67	0.06	1.60	1.29	0.000	3.33
DGI 307	4.91	2.88	0.02	1.71	1.84	0.001	2.67
DGI 75	5.13	2.76	0.01	1.86	1.86	0.000	2.76
DSL 104-1	4.90	2.95	0.03	1.66	1.48	0.000	3.31
DSU 4-7	4.52	2.47	0.02	1.83	1.39	0.000	3.26
IR36	3.89	1.78	0.00	2.18	0.45	0.000	8.63
IR55419-04	4.39	2.96	0.09	1.49	2.15	0.013	2.05
IR64	4.97	2.16	0.00	2.31	1.02	0.000	4.85
IR66873-R-11-1	4.94	2.10	0.00	2.35	0.66	0.000	7.47
IR67469-R-1-1	4.29	1.30	0.00	3.29	0.88	0.000	4.86
IR72667-16-1-B-B-3	4.38	2.79	0.06	1.57	1.82	0.002	2.40
IR74371-3-1-1	4.78	2.64	0.01	1.81	1.71	0.000	2.79
IR74371-46-1-1	4.68	2.65	0.01	1.77	1.83	0.000	2.55
IR74371-54-1-1	4.63	2.96	0.05	1.56	1.84	0.000	2.52
IR74371-70-1-1	5.10	2.92	0.01	1.75	1.87	0.000	2.72
IR74371-78-1-1	4.94	2.92	0.03	1.69	1.75	0.000	2.83
Kallurundaikar	4.51	2.65	0.04	1.70	1.96	0.003	2.30
Khiradhan	5.08	2.33	0.00	2.18	0.76	0.000	6.65
MTU 1010	4.79	2.59	0.01	1.85	1.43	0.000	3.36
NDR 1098-6	4.09	2.82	0.14	1.45	1.39	0.000	2.95
PM 1011	4.58	2.89	0.07	1.59	1.25	0.000	3.65
PMK 1	4.73	1.17	0.00	4.04	0.79	0.000	5.96
PMK 2	4.22	1.36	0.00	3.09	0.77	0.000	5.45
Poornima	4.00	2.55	0.05	1.57	1.68	0.001	2.39
R1027-2282-2-1	4.55	2.69	0.04	1.69	1.19	0.000	3.83
RF 5329	4.32	2.85	0.08	1.52	1.86	0.004	2.33
RR 272-21	4.33	2.66	0.05	1.63	1.26	0.000	3.43
Tripuradhan	4.55	2.88	0.04	1.58	2.03	0.002	2.24

The column p-value indicates the p-value for the test of difference between the mean under control and the mean under the respective stress level and indicates the probability that the observed difference between the means is due to chance.

**Table 2:** **Description of shallow rainfed lowland experimental sites**

Trial	Site description	Location	Target environment	Year of experimentation	Planned* level of stress	Assigned† level of stress	Trial mean yield (t/ha)
FZS05	Narendra Dev University of Agriculture and Technology (NDUAT), Faizabad, Uttar Pradesh	26°47^′^N, 82°12^′^E	Rainfed shallow lowland	2005	Severe	Control	5.56
FZM06				2006	Moderate	Moderate	3.38
FZS06				2006	Severe	Severe	1.30
FZC07				2007	Control	Control	4.09
FZM07				2007	Moderate	Moderate	3.22
FZS07				2007	Severe	Severe	2.17
HZC05	Central Rainfed Upland Rice Research Station (CRURRS), Hazaribag, Jharkhand	23°59^′^N, 82°25^′^E	Rainfed shallow lowland	2005	Control	Control	5.01
HZM05				2005	Moderate	Moderate	2.00
HZS05				2005	Severe	Control	3.92
HZM06				2006	Moderate	Severe	1.53
HZS06				2006	Severe	Moderate	1.04
HZC07				2007	Control	Control	4.58
HZM07				2007	Moderate	Moderate	3.26
HZS07				2007	Severe	Moderate	3.38
RPC05	Indira Gandhi Krishi Vishwavidyalaya (IGKV), Raipur, Chhattisgarh	21°14^′^N, 81°38^′^E	Rainfed shallow lowland	2005	Control	Control	3.83
RPM05				2005	Moderate	Severe	0.77
RPS05				2005	Severe	Moderate	2.85
RPC06				2006	Control	Control	4.75
RPM06				2006	Moderate	Moderate	2.63
RPS06				2006	Severe	Severe	1.34
RPM07				2007	Moderate	Moderate	3.25
RPS07				2007	Severe	Severe	1.87

* indicates the targeted level of stress in the field.

† indicates the achieved level of stress in the field.

### Statistical analysis

Yield is the final trait used to assess drought resistance of genotypes. The definition of drought resistance in terms of yield is linked to the drought stress scenarios presented by the TPE (Blum 2006). To study the yield performance of genotypes in a TPE, breeders evaluate their germplasm in multi-environment trials (METs). Testing under a range of environments, in particular drought stress levels, produces genotype x stress-level interactions. Multi-environment trials are often analyzed using mixed models (Annichiarico 2002,Smith et al. 2005). Data generated from METs are highly unbalanced due to variation in the entries tested from year to year, variation in the number of replicates at each site and variation in the sites that are included from year to year. In addition, drought-susceptible varieties may contribute to missing values when exposed to severe stress. Missing values, parameter estimation and prediction of genotype performance are effectively handled by mixed models (Piepho and Möhring 2006). When the mixed model and its error structure are complex it is worthwhile exploring different data transformations such that the transformation leads to a simpler analysis of the data (Piepho 2009).

Line means for each trial were first estimated fitting a mixed model by the REML algorithm taking lines as fixed and replicates and blocks within replicates as random. A conventional combined analysis of variance across years and sites was then done on log-transformed line means with all effects except stress level and genotype set to random. The performance *Y*_*lijk*_ of the i^th^ genotype at the j^th^ site within the k^th^ year at the s^th^ stress level is modeled as:

1 $$\begin{array}{ll} Y_{\text{lijk}}= & \mu +s_{i}+g_{i}+sg_{\text{li}}+br_{\text{jk}}+sbr_{\text{ljk}}+gbr_{\text{ijk}}\\ +e_{\text{lijk}} \end{array}$$

where *Y*_*ijk*_ is the log-transformed yield, *μ* is the overall mean, *s*_*i*_ is the effect of the s^th^ stress level, *g*_*i*_ is the effect of the i^th^ genotype, *s*_*gli*_ is the interaction of the i^th^ genotype at the l^th^ stress level, *b*_*rjk*_ is the interaction of the j^th^ site with the k^th^ year, *sbr*_*ljk*_ is the interaction of the l^th^ stress level, j^th^ site and k^th^ year, *gbr*_*ijk*_ is the interaction of the i^th^ genotype, j^th^ site and k^th^ year and *e*_*lijk*_ is a residual error. A separate residual error variance was fitted to each stress level. The least squares means for stress x genotype interactions were computed and pair wise comparisons involving the same genotype were made. The model was fitted using the MIXED procedure in SAS (Littell et al., 2006).

We propose the following drought yield index:

DYI=YiNSYiSGNSGS

where the subscripts *NS* and *S* refer to control and stress, respectively, *Yi* is the mean yield of a genotype on the untransformed scale, while *G* is the geometric mean across genotypes. Since geometric means are used, the index is commensurate with a multiplicative model on the original scale, which transforms to an additive model on the logarithmic scale. As *G*_*NS*_ and *G*_*s*_ are the same for all genotypes, DYI is the ratio $\frac{{\left(Y_{i}\right)}_{\text{NS}}}{{\left(Y_{i}\right)}_{S}}$that determines the ranking of genotypes. The ratios were calculated after back transforming the log-transformed means to the original scale. This ratio translates into a simple difference on the log-scale. The index to compute then just boils down to the mean difference for a genotype between a control and stressed environment. The mean yield index (MYI) is defined as the average of predicted means across the non-stress, moderate and severe stress levels. In addition the deviation of genotype means from that of the means of popular varieties IR64 and MTU1010 under all three stress levels were calculated.

Based on the mean grain yield across trials under non-stress, moderate and severe stress conditions, conventional drought tolerance indices, namely, the stress susceptibility index (SSI), stress tolerance (TOL), stress tolerance index (STI) and geometric mean productivity (GMP), were calculated for both moderate and severe stress conditions. Correlations between these indices and with the drought yield index (DYI) were evaluated using the CORR procedure of SAS.

## Results

### ANOVA

Results of a conventional analysis of variance of the log-transformed data for grain yield indicated highly significant genotype x stress-level interaction (p<0.0001). The REML estimates of variance components and fixed effects are presented in Table 3. For each category, based on the AIC values (not presented), it is concluded that the model with separate error variances for each stress level was better than the one with constant residual variance. At each stress level, i.e., moderate and severe, those entries with non-significant differences between means of the non-stress and the respective stress level are the drought-resistant genotypes.

**Table 3:** **REML estimates of variance and fixed effects parameters**

Source		
**Variance components**	**Estimate**	**p-value**
Site x year	0	-
Genotype x site x year	0.02	0.0005
Stress x site x year	0.11	0.003
Non-stress	0.02	<.0001
Moderate stress	0.08	<.0001
Severe stress	0.18	<.0001
**Fixed effect parameters**	**F- statistic**	**p-value**
Genotype	3.16	<.0001
Stress	17.93	0.0007
Stress x genotype	1.95	<.0001

The model shows separate variances for error for each stress level.

### Grain yield performance under non-stress, moderate stress and severe stress

Genotype mean yields range from 3.9 t ha^-1^ to 5.0 t ha^-1^ under non-stress, from 1.78 t ha^-1^ to 3.40 t ha^-1^ under moderate stress and from 0.45 t ha^-1^ to 2.19 t ha^-1^ under severe stress (Table 1). There was a high positive correlation (0.81) between the moderate stress and severe stress means and a low positive correlation between the non-stress yields with both moderate stress (0.07) and severe stress (0.004) yields. IR74371-70-1-1, DGI75, Khiradhan, IR64 and IR74371-78-1-1 were the best performers under non-stress. Of these, IR74371-70-1-1 and DGI75 yielded well at all stress levels. IR36 and PMK2 yielded low at all stress levels. IR74371-78-1-1 yielded well under non-stress and moderate stress and yield was reasonable under severe stress. ARB8 was the top performer under both moderate and severe stress, followed by IR55419-04 and ARB7. Khiradhan yielded very well under non-stress, but its yield declined by almost 50% under moderate stress and by 85% under severe stress. MTU 1010 was a moderate performer at all stress levels.

### Proposed drought yield index (DYI)

The drought yield index values for the moderate and severe stress cases appear in Table 1. In each case, the ratios of the non-stress to the stress means were relatively low for those genotypes with a non-significant difference between the means of the non-stress and the respective stress levels. Annada, ARB5, ARB7, ARB8 and NDR 1098–6 were tolerant of stress. Significant differences were noted between non-stress and severe stress category means for all entries under severe stress, which implies that the performance under non-stress and severe stress was considerably different. However, the entries that had relatively closer performance under non-stress and severe stress were ARB7, ARB8 and IR55419-04.

### Other drought tolerance indices

#### Moderate stress

ARB8 ranked first for most indices. With respect to STI, GMP, MRP and REI, the following seven lines were usually among the top seven ARB8, ARB3, DGI75, DGI307, DSL104-1, IR74371-70-1-1 and IR74371-78-1-1. The best performers for SSI and TOL were Annada, ARB2, ARB5, ARB7 and IR55419-04.

#### Severe stress

ARB8 ranked first for most indices. With respect to MP, STI, GMP, MRP and REI, the following lines were usually found within the top seven: DGI75, DGI307 and IR74371-70-1-1. The best performers for SSI and TOL were ARB5, ARB7, CB0-15-24 and IR55419-04 (Table 4).

**Table 4:** **Conventional drought response indices of genotypes under moderate and severe stress conditions**

Breeding line	Moderate stress								Severe stress							
	REI	MRP	STI	GMP	DYI	TOL	SSI	SSSI	REI	MRP	STI	GMP	DYI	TOL	SSI	SSSI
Annada	1.005 (25)	2.015 (24)	0.70 (25)	3.46 (25)	1.43 (3)	1.25 (3)	1.00 (3)	−0.122 (3)	1.083 (23)	3.09 (23)	0.33 (23)	2.62 (23)	2.49 (12)	2.48 (9)	0.91(12)	−0.062 (12)
ARB 2	1.085 (13)	2.092 (13)	0.75 (13)	3.59 (13)	1.45 (5)	1.35 (6)	1.03 (5)	−0.113 (5)	1.203 (17)	3.20 (17)	0.36 (21)	2.72 (21)	2.53 (14)	2.62 (13)	0.92 (14)	−0.056 (14)
ARB 3	1.214 (4)	2.206 (3)	0.84 (4)	3.80 (4)	1.61 (18)	1.82 (19)	1.25 (18)	−0.046 (18)	1.439 (6)	3.39 (7)	0.43 (8)	2.97 (8)	2.63 (16)	2.99 (19)	0.94 (16)	−0.04 (16)
ARB 4	0.980 (27)	1.983 (27)	0.68 (27)	3.41 (27)	1.57 (11)	1.54 (11)	1.20 (11)	−0.063 (11)	1.180 (19)	3.19 (19)	0.38 (18)	2.82 (18)	2.30 (7)	2.41 (7)	0.86 (7)	−0.095 (7)
ARB 5	1.022 (23)	2.031 (21)	0.71 (23)	3.49 (23)	1.44 (4)	1.29 (5)	1.02 (4)	−0.117 (4)	1.250 (13)	3.25 (13)	0.38 (19)	2.81 (19)	2.22 (5)	2.30 (4)	0.83 (5)	−0.111 (5)
ARB 6	1.054 (17)	2.054 (17)	0.73 (17)	3.54 (17)	1.72 (25)	1.94 (22)	1.38 (25)	−0.007 (25)	1.179 (20)	3.17 (20)	0.39 (16)	2.83 (16)	2.69 (18)	2.91 (17)	0.95 (18)	−0.032 (18)
ARB 7	1.070 (14)	2.080 (14)	0.74 (14)	3.57 (14)	1.41 (2)	1.24 (2)	0.97 (2)	−0.133 (2)	1.417 (7)	3.40 (5)	0.42 (10)	2.94 (10)	2.07 (3)	2.19 (1)	0.78 (3)	−0.143 (3)
ARB 8	1.261 (1)	2.265 (1)	0.88 (1)	3.87 (1)	1.33 (1)	1.12 (1)	0.83 (1)	−0.173 (1)	1.786 (1)	3.68 (1)	0.47 (1)	3.13 (1)	2.05 (2)	2.29 (3)	0.77 (2)	−0.149 (1)
Baranideep	1.115 (9)	2.114 (9)	0.77 (9)	3.64 (9)	1.60 (17)	1.74 (18)	1.25 (17)	−0.047 (17)	1.199 (18)	3.19 (18)	0.37 (20)	2.77 (20)	2.78 (21)	2.95 (18)	0.97 (21)	−0.02 (21)
CB 0-15-24	1.066 (15)	2.070 (15)	0.74 (15)	3.56 (15)	1.51 (8)	1.48 (10)	1.12 (8)	−0.085 (8)	1.388 (9)	3.37 (8)	0.43 (9)	2.97 (9)	2.18 (4)	2.37 (6)	0.82 (4)	−0.12 (4)
CB 2-458	0.939 (32)	1.941 (31)	0.65 (32)	3.34 (32)	1.94 (32)	2.26 (32)	1.61 (32)	0.060 (32)	0.852 (28)	2.85 (28)	0.32 (26)	2.55 (26)	3.32 (27)	3.25 (27)	1.06 (27)	0.039 (27)
DGI 237	0.960 (30)	1.961 (30)	0.67 (30)	3.38 (30)	1.60 (16)	1.61 (13)	1.25 (16)	−0.047 (16)	0.800 (30)	2.79 (31)	0.27 (30)	2.35 (30)	3.33 (28)	2.99 (20)	1.06 (28)	0.039 (28)
DGI 307	1.189 (7)	2.181 (7)	0.83 (7)	3.76 (7)	1.71 (24)	2.03 (26)	1.37 (24)	−0.010 (24)	1.414 (8)	3.37 (9)	0.44 (6)	3.00 (6)	2.67 (17)	3.08 (23)	0.95 (17)	−0.034 (17)
DGI 75	1.193 (6)	2.186 (6)	0.83 (6)	3.77 (6)	1.86 (31)	2.37 (33)	1.53 (31)	0.037 (31)	1.439 (5)	3.39 (6)	0.46 (2)	3.09 (2)	2.76 (20)	3.27 (28)	0.96 (20)	−0.023 (20)
DSL 104-1	1.215 (3)	2.205 (4)	0.84 (3)	3.80 (3)	1.66 (20)	1.95 (23)	1.32 (20)	−0.026 (20)	1.166 (22)	3.16 (22)	0.35 (22)	2.69 (22)	3.31 (26)	3.42 (33)	1.06 (26)	0.037 (26)
DSU 4-7	0.939 (31)	1.939 (32)	0.65 (31)	3.34 (31)	1.83 (29)	2.05 (27)	1.51 (29)	0.030 (29)	0.843 (29)	2.84 (29)	0.30 (27)	2.50 (27)	3.26 (25)	3.14 (24)	1.05 (25)	0.033 (25)
IR36	0.582 (36)	1.535 (36)	0.40 (36)	2.63 (36)	2.18 (34)	2.11 (28)	1.80 (34)	0.118 (34)	0.170 (39)	1.83 (39)	0.08 (39)	1.32 (39)	8.63 (39)	3.44 (34)	1.34 (39)	0.224 (39)
IR55419-04	1.093 (12)	2.097 (12)	0.76 (12)	3.60 (12)	1.49 (7)	1.44 (7)	1.08 (7)	−0.097 (7)	1.520 (3)	3.49 (2)	0.46 (4)	3.07 (4)	2.05 (1)	2.25 (2)	0.77 (1)	−0.149 (1)
IR64	0.900 (33)	1.917 (33)	0.63 (33)	3.27 (33)	2.31 (35)	2.81 (35)	1.88 (35)	0.142 (35)	0.598 (33)	2.58 (33)	0.25 (33)	2.26 (33)	4.85 (33)	3.94 (37)	1.20 (33)	0.133 (33)
IR66873-R-11-1	0.873 (34)	1.889 (34)	0.61 (34)	3.22 (34)	2.35 (36)	2.83 (36)	1.90 (36)	0.150 (36)	0.373 (35)	2.32 (35)	0.16 (38)	1.81 (38)	7.47 (38)	4.28 (38)	1.31 (38)	0.206 (38)
IR67469-R-1-1	0.470 (38)	1.442 (39)	0.33 (38)	2.36 (38)	3.29 (38)	2.98 (38)	2.31 (38)	0.272 (38)	0.268 (36)	2.01 (36)	0.18 (35)	1.94 (35)	4.86 (34)	3.41 (32)	1.20 (34)	0.134 (34)
IR72667-16-1-B	1.028 (22)	2.030 (22)	0.71 (22)	3.50 (22)	1.57 (12)	1.59 (12)	1.20 (12)	−0.061 (12)	1.213 (16)	3.21 (16)	0.39 (17)	2.83 (17)	2.40 (110	2.56 (12)	0.88 (11)	−0.076 (11)
IR74371-3-1-1	1.059 (16)	2.058 (16)	0.74 (16)	3.55 (16)	1.81 (28)	2.14 (29)	1.49 (28)	0.024 (28)	1.175 (21)	3.17 (21)	0.40 (14)	2.86 (14)	2.79 (22)	3.06 (21)	0.97 (22)	−0.019 (22)
IR74371-46-1-1	1.040 (19)	2.040 (19)	0.72 (19)	3.52 (19)	1.77 (27)	2.03 (25)	1.44 (27)	0.010 (27)	1.236 (15)	3.23 (15)	0.42 (12)	2.93 (12)	2.55 (15)	2.84 (16)	0.92 (15)	−0.052 (15)
IR74371-54-1-1	1.155 (8)	2.153 (8)	0.80 (8)	3.71 (8)	1.56 (10)	1.67 (15)	1.19 (10)	−0.064 (10)	1.374 (10)	3.34 (10)	0.41 (13)	2.92 (13)	2.52 (13)	2.80 (15)	0.91 (13)	−0.057 (13)
IR74371-70-1-1	1.255 (2)	2.240 (2)	0.87 (2)	3.86 (2)	1.75 (26)	2.18 (30)	1.42 (26)	0.003 (26)	1.522 (2)	3.45 (3)	0.46 (3)	3.09 (3)	2.72 (19)	3.23 (26)	0.96 (19)	−0.027 (19)
IR 74371-78-1-1	1.211 (5)	2.201 (5)	0.84 (5)	3.80 (5)	1.69 (22)	2.02 (24)	1.36 (22)	−0.015 (22)	1.370 (11)	3.33 (11)	0.42 (11)	2.94 (11)	2.83 (23)	3.19 (25)	0.98 (23)	−0.014 (23)
Kallurundaikar	1.006 (24)	2.006 (26)	0.70 (24)	3.46 (24)	1.70 (23)	1.86 (20)	1.37 (23)	−0.012 (23)	1.278 (12)	3.28 (12)	0.43 (7)	2.98 (7)	2.30 (8)	2.55 (11)	0.86 (8)	−0.095 (8)
Khiradhan	0.997 (26)	2.010 (25)	0.69 (26)	3.44 (26)	2.18 (33)	2.75 (34)	1.79 (33)	0.117 (33)	0.494 (34)	2.51 (34)	0.19 (34)	1.97 (34)	6.65 (37)	4.32 (39)	1.29 (37)	0.189 (37)
MTU 1010	1.042 (18)	2.043 (18)	0.72 (18)	3.52 (18)	1.85 (30)	2.21 (31)	1.53 (30)	0.037 (30)	0.964 (24)	2.97 (24)	0.33 (24)	2.62 (24)	3.36 (29)	3.37 (31)	1.06 (29)	0.042 (29)
NDR 1098-6	0.970 (28)	1.977 (28)	0.67 (28)	3.40 (28)	1.45 (6)	1.27 (4)	1.03 (6)	−0.113 (6)	0.871 (27)	2.88 (27)	0.27 (29)	2.38 (29)	2.95 (24)	2.70 (14)	1.00 (24)	0.001 (24)
PM 1011	1.112 (10)	2.111 (10)	0.77 (10)	3.64 (10)	1.59 (15)	1.69 (17)	1.22 (15)	−0.055 (15)	0.904 (26)	2.92 (26)	0.28 (28)	2.40 (28)	3.65 (31)	3.32 (29)	1.10 (31)	0.066 (31)
PMK 1	0.466 (39)	1.489 (37)	0.32 (39)	2.35 (39)	4.04 (39)	3.56 (39)	2.50 (39)	0.329 (39)	0.240 (38)	2.00 (37)	0.18 (36)	1.94 (36)	5.96 (36)	3.94 (36)	1.26 (36)	0.172 (36)
PMK 2	0.483 (37)	1.449 (38)	0.34 (37)	2.40 (37)	3.09 (37)	2.85 (37)	2.24 (37)	0.253 (37)	0.242 (37)	1.95 (38)	0.16 (37)	1.81 (37)	5.45 (35)	3.44 (35)	1.24 (35)	0.156 (35)
Poornima	0.856 (35)	1.853 (35)	0.59 (35)	3.19 (35)	1.57 (13)	1.46 (8)	1.21 (13)	−0.060 (13)	0.930 (25)	2.94 (25)	0.32 (25)	2.59 (25)	2.39 (10)	2.33 (5)	0.88 (10)	−0.079 (10)
R 1027-2282-2-1	1.028 (21)	2.028 (23)	0.71 (21)	3.50 (21)	1.69 (21)	1.86 (21)	1.36 (21)	−0.015 (21)	0.793 (31)	2.80 (30)	0.26 (32)	2.33 (32)	3.83 (32)	3.36 (30)	1.12 (32)	0.078 (32)
RF 5329	1.035 (20)	2.039 (20)	0.72 (20)	3.51 (20)	1.52 (9)	1.48 (9)	1.13 (9)	−0.082 (9)	1.245 (14)	3.24 (14)	0.39 (15)	2.83 (15)	2.33 (9)	2.47 (8)	0.86 (9)	−0.09 (9)
RR 272-21	0.967 (29)	1.968 (29)	0.67 (29)	3.39 (29)	1.63 (19)	1.67 (16)	1.28 (19)	−0.038 (19)	0.791 (32)	2.79 (32)	0.26 (31)	2.34 (31)	3.43 (30)	3.07 (22)	1.07 (30)	0.048 (30)
Tripuradhan	1.104 (11)	2.104 (11)	0.77 (11)	3.62 (11)	1.58 (14)	1.67 (14)	1.21 (14)	−0.058 (14)	1.455 (4)	3.42 (4)	0.45 (5)	3.04 (5)	2.24 (6)	2.52 (10)	0.84 (6)	−0.107 (6)

REI = relative efficiency index, MRP = mean relative performance, STI = stress tolerance index, GMP = geometric mean productivity, DYI = drought yield index, TOL = tolerance index, SSI = stress susceptibility index, SSSI = Schneider’s stress susceptibility index.

The numbers in parentheses indicate the genotype ranks for each index.

### Correlation between indices

There is a strong positive correlation between (a) REI, MRP, STI, GMP and MPI and among (b) DYI, TOL, SSI and SSSI whereas the correlation between indices in groups (a) and (b) is highly negative. Table 5 presents the correlations of the severe stress levels. The correlation between predicted means under moderate and severe stress levels was fairly high (0.69). A low positive correlation existed between predicted means of non-stress with that of moderate and severe stress levels respectively.

**Table 5:** **Correlation coefficients between drought yield index and common drought indices under severe drought stress**

	REI	MRP	STI	GMP	MPI	DYI	TOL	SSI	SSSI
REI	1								
MRP	0.99	1							
MRP	<.0001								
STI	0.98	0.97	1						
STI	<.0001	<.0001							
GMP	0.97	0.97	0.99	1					
GMP	<.0001	<.0001	<.0001						
MPI	0.88	0.89	0.92	0.92	1				
MPI	<.0001	<.0001	<.0001	<.0001					
DYI	−0.86	−0.88	−0.88	−0.92	−0.71	1			
DYI	<.0001	<.0001	<.0001	<.0001	<.0001				
TOL	−0.69	−0.67	−0.67	−0.67	−0.33	0.80	1		
TOL	<.0001	<.0001	<.0001	<.0001	0.0385	<.0001			
SSI	−0.90	−0.90	−0.90	−0.90	−0.68	0.93	0.91	1	
SSI	<.0001	<.0001	<.0001	<.0001	<.0001	<.0001	<.0001		
SSSI	−0.90	−0.90	−0.90	−0.90	−0.68	0.93	0.91	1	1
SSSI	<.0001	<.0001	<.0001	<.0001	<.0001	<.0001	<.0001	<.0001	

*REI* = relative efficiency index, *MRP* = mean relative performance, *STI* = stress tolerance index, *GMP* = geometric mean productivity, *MPI* = mean productivity index. *DYI* = drought yield index, *TOL* = tolerance index, *SSI* = stress susceptibility index, *SSSI* = Schneider’s stress severity index (modified).

In each cell, the number on top is the correlation coefficient and the number below is the p-value of observing the corresponding correlation coefficient.

### Mean yield index

ARB 8 recorded the highest MYI value of 3.34, followed by IR74371-70-1-1, DGI 75, DGI307, IR74371-78-1-1, and IR55419-04 with MYI values of 3.30, 3.25, 3.21, 3.20 and 3.17, respectively.

### Deviations from IR64 and MTU1010 means

The deviations of genotype means from means of the cultivated high-yielding check varieties (IR64 and MTU1010) are presented in Table 6. Two entries, IR74371-70-1-1 and DGI 75, showed positive deviations from IR64 under all three situations, whereas five entries, ARB3, DGI 75, DGI 307, IR74371-70-1-1 and IR74371-78-1-1, showed positive deviations from MTU 1010 under all three situations. However, many genotypes showed positive deviation in performance over both IR64 and MTU1010 under moderate and severe stress but only two genotypes, IR74371-70-1-1 and DGI 75, showed positive deviations from both IR64 and MTU1010 under all situations. ARB8, with the highest MYI however, did not show positive deviation from the performance of IR64 under irrigated situations because of its lower yield than IR64 under irrigated control situation. This indicates that the higher MYI of ARB8 is due to its significantly higher performance than IR64 under moderate and severe drought stress conditions.

**Table 6:** **Mean yield index**, **deviations from IR64 means and MTU 1010 means across non**-**stress moderate and severe stress levels**

ENTRY	Mean			MYI	Dev from IR64 mean			Dev from MTU 1010 mean		
	Control	Moderate	Severe		Control	Moderate	Severe	Control	Moderate	Severe
Annada	4.14	2.89	1.66	2.90	−0.83	0.73	0.64	−0.66	0.30	0.23
ARB 2	4.33	2.98	1.71	3.01	−0.64	0.83	0.69	−0.47	0.40	0.28
ARB 3	4.82	3.00	1.83	3.21	−0.15	0.84	0.80	0.02	0.41	0.40
ARB 4	4.27	2.73	1.86	2.95	−0.70	0.57	0.83	−0.52	0.14	0.43
ARB 5	4.19	2.90	1.89	2.99	−0.78	0.75	0.86	−0.61	0.32	0.46
ARB 6	4.64	2.70	1.73	3.02	−0.33	0.55	0.70	−0.16	0.12	0.30
ARB 7	4.24	3.00	2.05	3.09	−0.73	0.85	1.02	−0.56	0.42	0.62
ARB 8	4.47	3.35	2.19	3.34	−0.49	1.20	1.16	−0.32	0.77	0.76
Baranideep	4.61	2.87	1.66	3.05	−0.36	0.72	0.64	−0.18	0.29	0.23
CB 0-15-24	4.38	2.89	2.01	3.09	−0.59	0.74	0.99	−0.42	0.31	0.58
CB 2-458	4.65	2.40	1.40	2.82	−0.31	0.24	0.38	−0.14	−0.19	−0.03
DGI 237	4.28	2.67	1.29	2.74	−0.69	0.51	0.26	−0.51	0.08	−0.14
DGI 307	4.91	2.88	1.84	3.21	−0.06	0.72	0.81	0.12	0.29	0.41
DGI 75	5.13	2.76	1.86	3.25	0.16	0.61	0.84	0.34	0.18	0.43
DSL 104-1	4.90	2.95	1.48	3.11	−0.07	0.79	0.46	0.10	0.36	0.05
DSU 4-7	4.52	2.47	1.39	2.79	−0.45	0.31	0.36	−0.27	−0.12	−0.04
IR36	3.89	1.78	0.45	2.04	−1.08	−0.37	−0.57	−0.91	−0.81	−0.98
IR55419-04	4.39	2.96	2.15	3.17	−0.58	0.80	1.12	−0.40	0.37	0.72
IR64	4.97	2.16	1.02	2.72	0	0	0	0.17	−0.43	−0.40
IR66873-R-11-1	4.94	2.10	0.66	2.57	−0.03	−0.05	−0.36	0.14	−0.48	−0.77
IR67469-R-1-1	4.29	1.3	0.88	2.16	−0.68	−0.85	−0.14	−0.51	−1.28	−0.55
IR72667-16-1-B-B-3	4.38	2.79	1.82	3.00	−0.59	0.64	0.80	−0.41	0.20	0.39
IR74371-3-1-1	4.78	2.64	1.71	3.04	−0.19	0.48	0.69	−0.02	0.05	0.29
IR74371-46-1-1	4.68	2.65	1.83	3.05	−0.29	0.49	0.81	−0.12	0.06	0.41
IR74371-54-1-1	4.63	2.96	1.84	3.14	−0.34	0.81	0.81	−0.16	0.38	0.41
IR74371-70-1-1	5.10	2.92	1.87	3.30	0.13	0.77	0.85	0.31	0.34	0.44
IR74371-78-1-1	4.94	2.92	1.75	3.20	−0.03	0.76	0.72	0.14	0.33	0.32
Kallurundaikar	4.51	2.65	1.96	3.04	−0.46	0.50	0.94	−0.28	0.07	0.53
Khiradhan	5.08	2.33	0.76	2.73	0.12	0.18	−0.26	0.29	−0.25	−0.66
MTU 1010	4.79	2.59	1.43	2.94	−0.17	0.43	0.40	0	0	0
NDR 1098-6	4.09	2.82	1.39	2.77	−0.88	0.66	0.36	−0.70	0.23	−0.04
PM 1011	4.58	2.89	1.25	2.91	−0.39	0.73	0.23	−0.22	0.30	−0.17
PMK 1	4.73	1.17	0.79	2.23	−0.24	−0.98	−0.23	−0.06	−1.42	−0.63
PMK 2	4.22	1.36	0.77	2.12	−0.75	−0.79	−0.25	−0.58	−1.22	−0.65
Poornima	4.00	2.55	1.68	2.74	−0.97	0.39	0.65	−0.79	−0.04	0.25
R 1027-2282-2-1	4.55	2.69	1.19	2.81	−0.42	0.53	0.16	−0.24	0.10	−0.24
RF 5329	4.32	2.85	1.86	3.01	−0.64	0.69	0.83	−0.47	0.26	0.43
RR 272-21	4.33	2.66	1.26	2.75	−0.64	0.50	0.24	−0.47	0.07	−0.17
Tripuradhan	4.55	2.88	2.03	3.16	−0.42	0.73	1.01	−0.24	0.30	0.61
Overall mean	4.54	2.62	1.54							

*MYI*= Mean yield index.

## Discussion

In our study, the significance of genotype x stress-level interaction was first investigated using an appropriate mixed model. The effect of stress level and genotypes was considered fixed because these were the only levels and genotypes of interest. The objective is to test the significance of genotype x stress interaction across all potential drought-prone sites and years. Interest is not specifically in those sites and years but the target population from which they were drawn. Therefore, years and sites were considered as random. When treatments are evaluated at different stress levels, they tend to have different levels of error variation. Therefore, heterogeneity of error variances at different stress levels was incorporated. The least squares means for the stress x genotype interaction were computed after appropriately accounting for all potential sources of variation. Then, pair wise stress-level comparison of means (non-stress vs moderate stress and non-stress vs severe stress) involving the same entry was carried out to look for those entries with statistically non-significant differences between means across the two stress levels.

At the reproductive stage, yield reduction in rice is significant even with a moderate stress (Verulkar et al. 2010). However, this is lower than that under severe drought at the reproductive stage. Most studies point out a negative relationship between yield potential (yield under non-stress) and yield under severe stress, but varieties with high yield potential will generally have an advantage over varieties with lower yield potential under moderate drought stress. Hence, our study aims at calculating the response index under both moderate and severe stress to identify varieties that are tolerant of both moderate and severe drought. The proposed drought yield index (DYI) is similar to ones proposed by previous authors (Fernandez 1992,Araghi and Assad 1998) based on the ratio of means under non-stress to the respective stress level (moderate or severe). However, the main advantage of the current method is that DYI is calculated as a linear function of parameter estimates within the framework of a mixed model after accounting for a significant genotype x stress-level interaction across environments and it therefore provides more precise statistical inference, which was not the case with the earlier ratio-based measures.

A high positive correlation between the moderate and severe stress means indicates that the entries that performed well under moderate stress also performed well under severe stress. STI and GMP identified DGI75 (5.13, 2.76, 1.86 t/ha) and IR74371-70-1-1 (5.10, 2.92, 1.87 t/ha) among the top three stable performers. These two, along with ARB8 (4.47, 3.35, 2.19 t/ha) and IR55419-04 (4.39, 2.96, 2.15 t/ha), were the top five. This implies that STI and GMP may be useful in identifying entries that yield well under non-stress and yield reasonably well under severe stress. The proposed DYI, TOL and SSI favor genotypes with good yield under stress. For these indices, DGI75, DGI307 or IR74371-70-1-1 do not stand out among the top performers. These indices are therefore more useful for identifying stress-tolerant genotypes that perform well in stress environments.

ARB8 is again the top performer in the case of MRP and REI as well. This is because these indices are respectively the sum and product of two ratios, (i) genotype control mean/overall control mean and (ii) genotype severe-stress mean/overall severe-stress mean. The index values are inflated if either (i) or (ii) is higher. If (ii) is high, the entry enters the set of top performers though the performance under non-stress is not the topmost and vice versa. So, these indices are not very effective in distinctively discriminating entries that perform well under both non-stress and stress.

For areas where severe stress is a recurrent phenomenon, selection of genotypes with high DYI can be useful. However, selection based on DYI may lead to the identification of genotypes with high yield in moderate or severe drought stress but not very high yield or yield equivalent to that of current cultivated varieties under normal irrigated situations. MYI that considers the performance of genotypes under different situations ranging from irrigated control to moderate drought stress and severe drought stress provides better opportunities for breeders to select genotypes with good performance under all situations. The careful study of the deviation in performance of 39 genotypes from two popular varieties (IR64 and MTU1010) revealed that the performance of only two genotypes (IR74371-70-1-1 and DGI 75) showed a positive deviation over the performance of both popular varieties under the three conditions. Even though genotypes such as ARB 8, DGI307, IR55419-04, and IR74371-78-1-1 showed a high MYI, they did not show a positive deviation in their performance under all situations due to their lower yield than IR64 under irrigated control situations. For areas with a variable rainfall pattern and with equal probability of occurrence of normal rainfall, moderate stress or severe stress situations, breeders should combine MYI with a positive deviation in the performance of genotypes under all situations in order to select a variety with better performance under all situations over current varieties. In fact, even for highly drought-prone areas, combinations of DYI and deviations in the performance of genotypes from that of current varieties will enable breeders to select genotypes with yield very near that of current cultivated varieties under irrigated normal situations in addition to their higher yield under moderate and severe drought situations.

## Conclusions

Many authors have reported that selection of stable genotypes should be based on a combination of indices. For regions with severe drought problems and higher frequency of drought occurrence, our study indicated that selection based on DYI will result in the identification of lines with significantly higher performance over current cultivated varieties under moderate to severe drought at the expense of slightly lower yield under normal irrigated situations. For regions where moderate to severe drought occurs once in three to four years and normal rainfall persists over other years, selection of genotypes based on a combination of MYI and a positive response over existing high-yielding varieties under all situations will enable breeders to select genotypes with higher yield over current varieties under all situations. From among 39 genotypes, the combination of MYI and deviation identified IR74371-70-1-1 and DGI 75 as two promising high-yielding drought-tolerant cultivars.
